# Exercise reverses age‐related vulnerability of the retina to injury by preventing complement‐mediated synapse elimination via a BDNF‐dependent pathway

**DOI:** 10.1111/acel.12512

**Published:** 2016-09-09

**Authors:** Vicki Chrysostomou, Sandra Galic, Peter van Wijngaarden, Ian A. Trounce, Gregory R. Steinberg, Jonathan G. Crowston

**Affiliations:** ^1^ Centre for Eye Research AustraliaRoyal Victorian Eye and Ear HospitalUniversity of Melbourne32 Gisborne StreetEast MelbourneVic.3002Australia; ^2^ St. Vincent's Institute of Medical Research and Department of MedicineUniversity of Melbourne41 Victoria ParadeFitzroyVic.3065Australia; ^3^ Division of Endocrinology and MetabolismDepartment of MedicineMcMaster University1280 Main Street WestHamiltonONL8S 4L8Canada; ^4^ Department of Biochemistry and Biomedical SciencesMcMaster University1280 Main Street WestHamiltonONL8S 4L8Canada

**Keywords:** anti‐aging, brain‐derived neurotrophic factor, exercise, mouse model, neuroprotection, retinal ganglion cell

## Abstract

Retinal ganglion cells (RGCs) become increasingly vulnerable to injury with advancing age. We recently showed that this vulnerability can be strongly modified in mice by exercise. However, the characteristics and underlying mechanisms of retinal protection with exercise remain unknown. Hence, the aim of this study was to investigate cellular changes associated with exercise‐induced protection of aging retinal cells and the role of local and peripheral trophic signalling in mediating these effects. We focussed on two molecules that are thought to play key roles in mediating beneficial effects of exercise: brain‐derived neurotrophic factor (BDNF) and AMP‐activated protein kinase (AMPK). In middle‐aged (12 months old) C57BL/6J mice, we found that exercise protected RGCs against dysfunction and cell loss after an acute injury induced by elevation of intra‐ocular pressure. This was associated with preservation of inner retinal synapses and reduced synaptic complement deposition. Retinal expression of BDNF was not upregulated in response to exercise alone. Rather, exercise maintained BDNF levels in the retina, which were decreased postinjury in nonexercised animals. Confirming a critical role for BDNF, we found that blocking BDNF signalling during exercise by pharmacological means or genetic knock‐down suppressed the functional protection of RGCs afforded by exercise. Protection of RGCs with exercise was independent of activation of AMPK in either retina or skeletal muscle. Our data support a previously unidentified mechanism in which exercise prevents loss of BDNF in the retina after injury and preserves neuronal function and survival by preventing complement‐mediated elimination of synapses.

## Introduction

The prevalence and incidence of glaucoma, a neurodegenerative disease characterized by the selective loss of retinal ganglion cells (RGCs), increases almost exponentially with age across all major populations (Quigley & Broman, 2006). While age has traditionally thought to be a nonmodifiable risk factor for disease, emerging evidence suggests this may not be the case. In support, we recently discovered that exercise in middle‐aged mice, in the form of daily swimming, robustly protected RGCs against age‐related functional loss and signs of stress after an acute injury (Chrysostomou *et al*., 2014). The effect was so potent that exercised 12‐month‐old mice responded to injury in a similar manner to young nonexercised 3‐month‐old mice. These data provide compelling evidence that exercise can reverse negative impacts of aging in RGCs and modify their response to injury.

However, several key questions remain. Foremost, the biological mechanisms underlying protection of aging retinal cells with exercise are currently unknown. Neurotrophins and growth factors such as brain‐derived neurotrophic factor (BDNF) and insulin‐like growth factor‐1 are thought to act as broad signalling molecules to mediate beneficial effects of exercise in other regions of the CNS (Carro *et al*., 2001; Vaynman *et al*., 2004; Gomez‐Pinilla *et al*., 2008; Lawson *et al*., 2014), but it is not clear whether similar factors are involved in protecting cells within the retina or if/how they are altered with age.

It is also unclear how these signalling molecules affect cell structure or function in target tissues in order to confer protective effects. In the case of BDNF, it has been suggested that neuroprotective mechanisms may involve modulation of synaptic plasticity and energy metabolism (Vaynman *et al*., 2006).

A further unknown is whether exercise has a direct effect on the retina or whether protection is conferred indirectly via signals derived from the periphery. Accumulating evidence suggests that pleiotropic effects of exercise in different organ systems are mediated by myokines, factors produced and secreted by skeletal muscle that can trigger protective pathways in distal tissues (Pedersen *et al*., 2007; Wrann *et al*., 2013). This provides a promising candidate pathway for linking exercise with beneficial effects in the CNS. While the mechanisms underlying myokine signalling are still being elucidated, reports suggest a critical role for the metabolic sensor AMP‐activated protein kinase (AMPK) in stimulating the expression, release and action of these muscle‐derived factors (Lally *et al*., 2015; Lee *et al*., 2015). AMPK activation also mediates a range of other metabolic responses to exercise such as energy control, glucose uptake and fatty acid oxidation (Steinberg & Kemp, 2009).

The current study has investigated cellular changes and signalling pathways that underlie protection of RGCs with exercise. We focussed on two molecules that are centrally involved in metabolic responses to exercise: BDNF and AMPK. We investigated the role of both local and peripheral BDNF and AMPK signalling pathways in mediating the protective effects of exercise on the mouse retina after an acute injury. Identifying the nature, source and action of molecular mediators of exercise‐induced protection will inform the development of novel therapies to modify the response of aging cells to injury and protect against age‐related diseases such as glaucoma.

## Results

### Exercise initiated 24 h postinjury protects RGCs against age‐related functional loss

**Figure 1:**
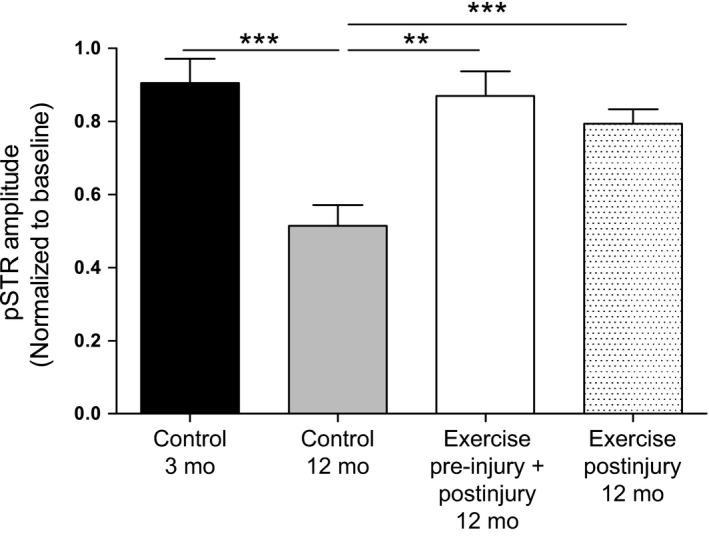
Exercise prevents age‐related RGC dysfunction after injury. The electroretinogram (ERG) was recorded serially in 3‐ and 12‐month‐old C57BL/6J mice, 1 day before (baseline) and 7 days after pressure‐induced injury to the inner retina. Amplitudes of the pSTR component of the ERG were used as a measure of RGC function and are presented as relative changes from baseline. Function was also compared between 12‐month‐old mice that were exercised both before and after injury (5 weeks before, 7 days after) and mice that were only exercised after injury. Data are presented as mean ± SEM;* n* = 10 per group; **P < 0.01, ***P < 0.001 by Student's *t*‐test.

In the current study, we utilized a well‐characterized nonischaemic injury to the inner retina in the form of short‐term elevation of intra‐ocular pressure (IOP) (Chrysostomou *et al*., 2014; Crowston *et al*., 2015). Using the electroretinogram (ERG) to measure *in vivo* RGC function, we found that the response to this injury was strongly age dependent, confirming previous reports (Kong *et al*., 2012; Chrysostomou *et al*., 2014). Whereas middle‐aged (12 month old) mice exhibited a 40–50% reduction in amplitudes of the positive scotopic threshold response (pSTR) at 7 days postinjury, almost full functional recovery was observed in younger 3‐month‐old mice (Fig. 1). This age‐related vulnerability was almost completely reversed by exercising mice for 5 weeks before and 7 days after injury, supporting our earlier study (Chrysostomou *et al*., 2014). To address the potential confounder that cardiovascular and other systemic factors present in exercised mice might lessen the injury load, we next investigated the effect of exercise that was initiated 24 h after injury in 12‐month‐old mice. We found that exercise for 7 days after injury provided an identical degree of functional protection (Fig. 1) and have therefore used this postinjury exercise regime in 12‐month‐old mice for all experiments presented here.

### Exercise prevents inner retinal synaptic loss after injury

**Figure 2:**
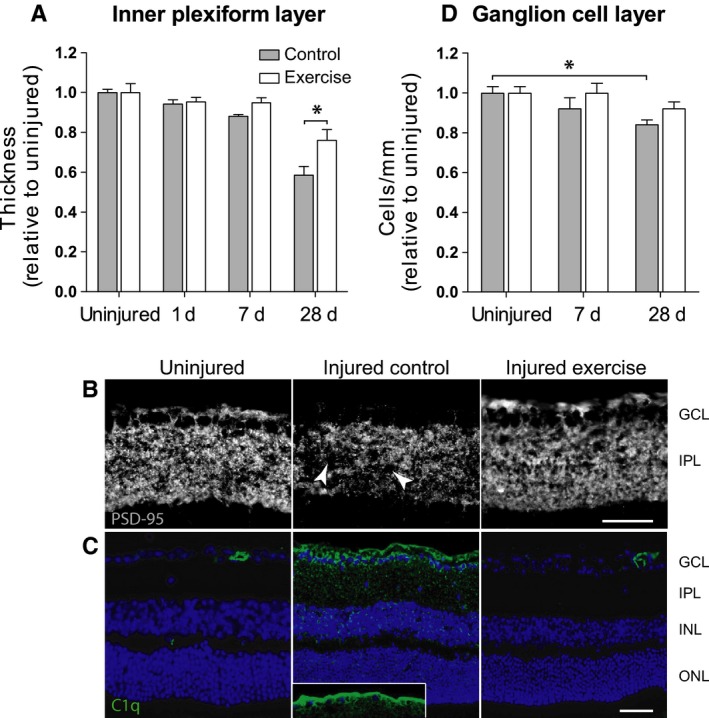
Exercise prevents inner retinal synaptic loss and complement deposition after injury and promotes cell survival. Injury to RGCs was induced in 12‐month‐old C57BL/6J mice by elevation of intra‐ocular pressure before equal numbers of animals were assigned to control or exercise conditions. (A) Thickness of the inner plexiform layer (IPL) was quantified on retinal cross sections in the month following injury and compared between mice under control conditions and mice that were exercised for 7 days postinjury. Data are normalized to uninjured eyes and presented as mean ± SD (*n* = 9); *P < 0.05 by Student's *t*‐test. (B) Injured eyes from nonexercised and exercised mice were assessed using immunohistochemistry 7 days after injury. Representative images of retinal cryosections stained for postsynaptic protein‐95 (PSD‐95) showing focal synapse loss (*arrows*) in the IPL. Scale bar = 50 μm; GCL: ganglion cell layer. (C) Immunohistochemical labelling for complement protein C1q (*green*) in retinal cryosections counterstained with Hoechst (*blue*). Inset shows C1q labelling of RGC axons in the nerve fibre layer. Scale bar = 50 μm. ONL: outer nuclear layer; INL: inner nuclear layer. (D) Cell survival up to 1 month after injury was assessed by counting soma in the RGC layer of Hoechst‐stained cross sections. Data are normalized to uninjured eyes and presented as mean ± SD (*n* = 9); *P < 0.05 by one‐way ANOVA.

To investigate structural changes that may account for functional protection of RGCs in 12‐month‐old mice with exercise, we assessed the integrity of the synaptic layers in retinal cross sections. We identified a gradual and significant thinning of the inner plexiform layer (IPL) in retinas from control (nonexercised) animals in the month following injury (Fig. 2A). The IPL contains synaptic connections between bipolar interneurons of the mid‐retina and RGCs of the inner retina. Relative to uninjured fellow eyes, IPL thickness was significantly reduced to 94 ± 5.2% at 1 day postinjury and continued to thin in a time‐dependent manner, reducing to 88 ± 2.6% at 7 days and 59 ± 1.2% by day 28. In contrast, there was no significant change in IPL thickness in retinas taken from middle‐aged mice that had exercised for 7 days postinjury at either 1 or 7 days (Fig. 2A). Between 7 and 28 days, animals in the exercise group stopped exercising and were switched to control conditions. During this time, when exercise had ceased, the IPL of injured eyes reduced in thickness compared to uninjured eyes, but this thinning was less severe than that seen in control animals that had not exercised at all. In a direct comparison, the IPL at day 28 was significantly (*P* < 0.05) thicker in injured eyes of exercised animals vs. injured eyes of nonexercised animals (76 ± 0.5% and 58 ± 0.4%, respectively). Given that our previous work demonstrated protection against RGC dysfunction in exercised animals following injury (Chrysostomou *et al*., 2014), these new findings results suggest that exercise promotes inner retinal function through maintenance of synapses in the IPL.

To further investigate synaptic integrity in the IPL, RGC dendrites were immunolabelled for postsynaptic density protein‐95 (PSD‐95) on tissue cross sections. Immunofluorescence for PSD‐95 was prominent and uniform throughout the IPL of uninjured retinas (Fig. 2B), reflecting dense synaptic connections between bipolar cells and RGCs. In response to injury, PSD‐95 labelling became patchy and less intense. It has previously been reported that this change in PSD‐95 distribution is indicative of focal synapse loss (Stevens *et al*., 2007). In retinas from animals that had exercised for 7 days postinjury, PSD‐95 immunolabelling throughout the IPL was uniform and similar in intensity to that seen in uninjured eyes (Fig. 2B). This result supports the hypothesis that RGC injury causes loss or retraction of inner retinal synapses and that exercise is able to mitigate these effects.

### Exercise reduces synaptic complement deposition after injury

Elimination of neuronal synapses can result from complement activation and microglia‐mediated clearance (Stevens *et al*., 2007). We therefore next investigated whether exercise was associated with a reduction in complement activation in the IPL. Using immunohistochemistry, we investigated retinal expression and localization of C1q, the initiator of the classical complement cascade and a critical point of regulation in complement‐mediated synaptic pruning (Bialas & Stevens, 2013). As shown in Fig. 2C, we observed little C1q expression on immunolabelled cross sections from uninjured retinas. Some immunoreactivity was seen within the inner retinal vasculature, presumably due to the presence of complement components in serum. In response to injury, there was a pronounced increase in punctate C1q staining in the IPL, corresponding in time and location to the focal synaptic loss we detected with PSD‐95. C1q immunolabelling also concentrated in the RGC layer of injured retinas and was present along the nerve fibre layer of the retina, where RGC axons are located (Fig. 2C, inset). In marked contrast, retinas taken from mice that had exercised postinjury showed little C1q immunoreactivity. Low levels of focal punctate C1q staining was seen in the ganglion cell layer and inner laminae of the IPL in three of six exercised retinas but was entirely absent from the remaining three. Confirming previous reports, mid‐ and outer retinal layers were not complement immunoreactive in any sample analysed (Ding *et al*., 2012). Collectively, the results of our PSD‐95 and C1q staining suggest that the complement cascade mediates inner retinal synapse loss after injury and that amelioration of complement activation and/or deposition by exercise prevents this synaptic elimination.

### Exercise reduces RGC loss after injury

We next determined whether improved RGC functional recovery with exercise was associated with greater cell survival. To do so, we quantified soma in the ganglion cell layer (GCL) of retinal cross sections collected 7 and 28 days postinjury using a technique previously described (Howell *et al*., 2012). Low levels of cell loss were detected in injured eyes of control (nonexercised) animals. By 28 days, there was a significant 16 ± 2.1% (*P* < 0.01) reduction in somas in the GCL (Fig. 2D). Exercise for 7 days postinjury prevented cell loss; there was no significant change in cell number at 28 days postinjury in eyes from animals that had been exercised. It is interesting to note that there was a small but nonsignificant reduction (8.0 ± 6.0%; *P* = 0.13) in GCL somas between 7 and 28 days, the time during which animals had stopped exercising.

There was no change in the thickness of the outer or middle nuclear layers of the retina with injury or exercise at any time point examined, indicating no loss of photoreceptors or mid‐retinal neurons (data not shown). Furthermore, there was no thinning of the outer plexiform layer in the 28 days following injury in either exercise or control retinas, suggesting that synaptic connections between photoreceptors and bipolar cells were unaffected. These findings confirm the specificity of this injury model to the inner retina (Crowston *et al*., 2015).

### Exercise maintains retinal BDNF levels postinjury

**Figure 3:**
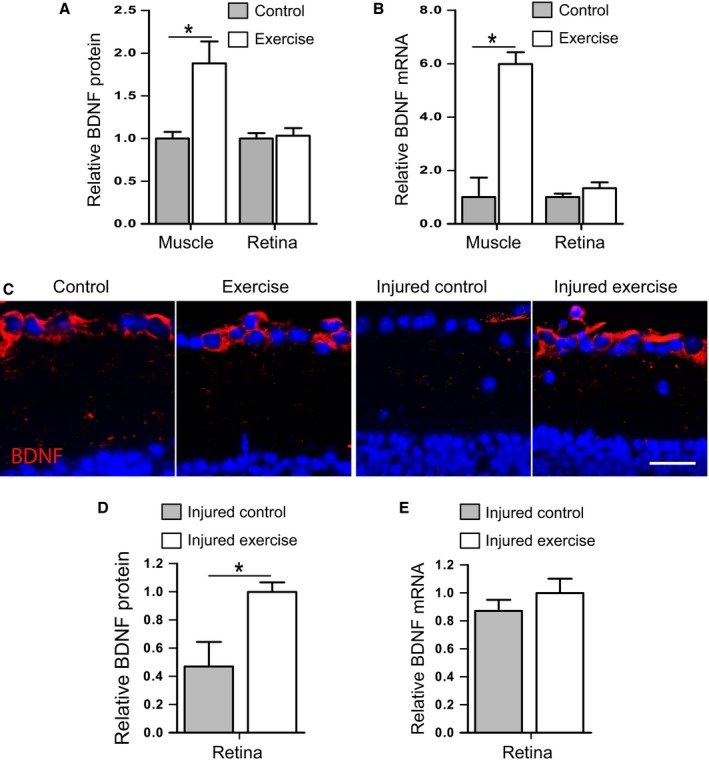
BDNF protein and gene expression in response to exercise and retinal injury. Levels of total BDNF protein (A) and BDNF mRNA (B) were measured in soleus muscle and retina of 12‐month‐old C57BL/6J mice that were exercised daily by swimming for 7 consecutive days. (C) Retinal protein expression of BDNF was also assessed by immunohistochemistry. Representative images show BDNF labelling (*red*) in the RGC layer of retinal cryosections. Scale bar = 50 μm. Injured eyes from nonexercised and exercised 12‐month‐old C57BL/6J mice were also assessed for BDNF protein (C,D) and gene (E) expression. Data are expressed relative to control (A,B) or exercise (D,E) values; mean ± SEM;* n* = 10; *P < 0.05 by Student's *t*‐test.

The neurotrophin BDNF has been reported to play a central role in exercise‐induced changes in the brain (reviewed in Pedersen *et al*., 2009). We next investigated whether BDNF was critical to exercise‐mediated protection in the retina. First, we measured local (retinal) and peripheral (skeletal muscle and blood) BDNF protein and gene expression after exercise in mice that were not exposed to RGC injury. Total BDNF protein was significantly increased (2‐fold) in soleus muscle following 7 days of exercise (Fig. 3A; *P* < 0.05). This was accompanied by a significant elevation of muscle BDNF mRNA expression (Fig. 3B; *P* < 0.05) and confirms previous reports (Dupont‐Versteegden *et al*., 2004; Ogborn & Gardiner, 2010). In contrast to skeletal muscle, retinal tissue showed no detectable increase in either total BDNF protein content or BDNF mRNA levels following exercise (Fig. 3A,B). We further analysed BDNF protein expression and location in the retina using immunohistochemical labelling. In retinas from nonexercised mice, BDNF immunoreactivity was most prominent in somas of the ganglion cell layer (Fig. 3C), concurring with previous reports (Vecino *et al*., 2002; Seki *et al*., 2003). The distribution and intensity of retinal BDNF labelling by immunohistochemistry was not different in exercised mice compared with nonexercised mice. These observations suggest that exercise *per se* does not alter BDNF content in the retina. We were unable to detect BDNF protein levels in serum or plasma of mice using a commercially available ELISA kit, concurring with previous reports (Rasmussen *et al*., 2009; Klein *et al*., 2011).

We next looked at retinal BDNF expression in mice that had been exposed to RGC injury and found a dramatic reduction in expression in the inner retina 7 days after injury. As shown in Fig. 3C, BDNF immunoreactivity on retinal cryosections was almost entirely absent from the RGC layer of injured retinas. In marked contrast, retinas taken from animals that had exercised for 7 days postinjury displayed strong BDNF expression in somas of the RGC layer, with a similar pattern to uninjured eyes (Fig. 3C). Quantification of total BDNF protein content in injured retinas confirmed significantly lower levels in control mice compared with exercised mice (Fig. 3D; *P* < 0.05). Interestingly, despite lower levels of BDNF protein in control retinas compared with exercised injured retinas, BDNF mRNA levels were not different between groups (Fig. 3E).

### BDNF signalling is critical for functional protection conferred by exercise

**Figure 4:**
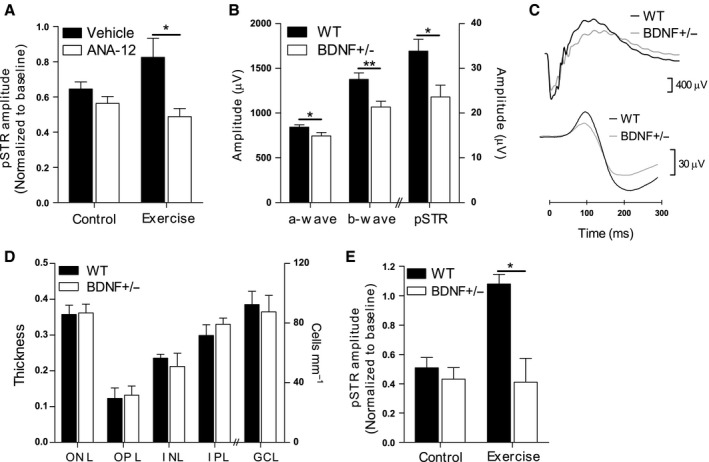
Exercise‐mediated functional protection is critically dependent on BDNF signalling. (A) For 7 days following retinal injury, 12‐month‐old C57BL/6J mice under control and exercise conditions received daily intraperitoneal injections of the TrkB antagonist ANA‐12 (*n* = 7). The ERG was used to record inner retinal function before (baseline) and 7 days after injury and results are presented as relative changes in pSTR amplitude from baseline. (B) Retinal function was assessed in 12‐month‐old mice carrying a heterozygous deletion of the BDNF gene (BDNF+/−) and compared to that of wild‐type (WT) mice (*n* = 12). Maximum amplitudes of ERG responses derived from photoreceptors (a‐wave), bipolar cells (b‐wave) and RGCs (pSTR) are shown. (C) Representative ERG waveforms recorded from a WT and BDNF+/− mouse demonstrating the a‐wave and b‐wave (top traces) and pSTR (bottom traces) components. (D) Cellular and synaptic layers were quantified on retinal cross sections taken from 12‐month‐old WT and BDNF+/− mice (*n* = 6). ONL: outer nuclear layer; OPL: outer plexiform layer; INL: inner nuclear layer; IPL: inner plexiform layer; GCL: ganglion cell layer. (E) Inner retinal injury was induced in BDNF+/− and WT mice before equal numbers of animals of each genotype were assigned to control or exercise conditions for 7 days (*n* = 6). Amplitudes of the pSTR component of the ERG were measured in all mice before (baseline) and 7 days after injury and results are presented as relative changes from baseline. All data are presented as mean ± SEM; *P < 0.05, **P < 0.01 by Student's *t*‐test.

We used two approaches to test whether the protective effects of exercise after retinal injury are dependent on BDNF: pharmacological receptor blockade and a knock‐out mouse model. ANA‐12 is a highly selective antagonist of the BDNF receptor TrkB that readily crosses the blood–brain barrier after systemic administration (Cazorla *et al*., 2011). Figure 4A shows that treatment of mice with ANA‐12, at a dose known to decrease TrkB activity in the mouse brain without affecting neuronal survival (Cazorla *et al*., 2011), completely suppressed the functional protection conferred by exercise after injury. Using the ERG to record *in vivo* function, we found that amplitudes of the positive scotopic threshold response (pSTR), which is derived predominantly from RGCs (Saszik *et al*., 2002), were reduced significantly (48.9 ± 4.5% of baseline values; *P* < 0.05) in exercised mice that were injected daily with ANA‐12. In contrast, pSTR amplitudes were preserved at 82.5 ± 10% of baseline values in vehicle‐treated exercised mice. ANA‐12 treatment suppressed exercise‐induced protection to the point that pSTR amplitudes after injury were statistically indistinguishable from those seen in nonexercised mice (Fig. 4A).

To further test whether exercise‐induced retinal protection is mediated by BDNF signalling, we conducted experiments with mice carrying a heterozygous deletion of the BDNF gene (BDNF+/−). These animals have a 40–60% reduction in BDNF protein and mRNA in retina and brain compared with wild‐type (WT) mice (Wilson *et al*., 2007). As shown in Fig. 4B,C, analysis of ERG responses prior to injury revealed that baseline retinal function was diminished in BDNF+/− mice compared with WT littermates. Maximum amplitudes of the photoreceptor‐derived a‐wave were slightly but significantly smaller in BDNF+/− animals (12% reduction; *P* < 0.05), while bipolar cell‐derived b‐waves were reduced to a greater extent (22% reduction; *P* < 0.01), confirming previous reports (Wilson *et al*., 2007). The greatest functional deficit was seen in responses derived from RGCs as reflected by a 31% reduction (*P* < 0.05) in pSTR amplitudes in BDNF+/− mice. Quantification of photoreceptors, bipolar cells and RGCs in retinal cross sections revealed no difference in cell number between BDNF+/− mice and WT mice (Fig. 4D), indicating that the functional deficit in BDNF+/− animals was not a consequence of substantial cell loss. Thickness of the outer and inner synaptic layers of the retina was also similar between genotypes.

Although 12‐month‐old BDNF+/− mice showed reduced pSTR amplitudes at baseline, their functional response to pressure‐induced injury was indistinguishable from WT mice. One week after injury, pSTR amplitudes were reduced to 40–50% of initial values in both BDNF+/− mice and WT mice (Fig. 4E). We next compared the protective effects of exercise in BDNF+/− vs. WT mice. As shown in Fig. 4E, WT mice that were exercised for 7 days postinjury were completely protected against pSTR functional loss. However, BDNF+/− mice that underwent the same postinjury exercise regime were not protected, as demonstrated by a reduction in pSTR amplitudes to 41 ± 11% of initial values. These results suggest that a full genetic complement of BDNF is critical for exercise‐induced protection of RGCs.

### AMPK activation in retina is not critical for functional protection with exercise

**Figure 5:**
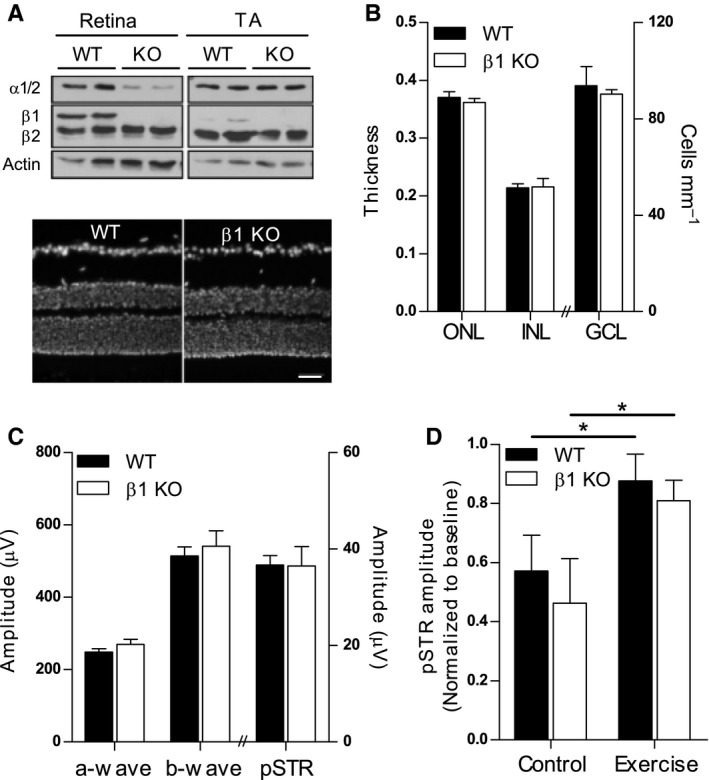
AMPK activation in retina is not required for protection from injury with exercise. (A) Expression of AMPK α and β subunits was assessed in retina and tibialis anterior (TA) muscle of 12‐month‐old mice with a homozygous deletion of the β1 subunit of AMPK (KO) and wild‐type (WT) littermates. (B) Nuclear‐stained retinal cryosections showing gross retinal structure in KO and WT mice. Scale bar = 50 μm. (C) Survival of retinal neurons was quantified by measuring thicknesses of the outer and inner nuclear layers (ONL, INL) and by counting the number of soma in the ganglion cell layer (GCL) of nuclear‐stained retinal cross sections (*n* = 10). (D) Electroretinography was used to assess the function of photoreceptors (a‐wave), bipolar cells (b‐wave) and RGCs (pSTR) in 12‐month‐old WT and KO mice (*n* = 12). Maximum absolute amplitudes of ERG responses are shown. (E) Inner retinal injury was induced in WT and KO mice before equal numbers of animals of each genotype were assigned to control or exercise conditions for 7 days (*n* = 6). Amplitudes of the pSTR component of the ERG were measured in all mice before (baseline) and 7 days after injury and results are presented as relative changes from baseline. All data are presented as mean ± SEM; *P < 0.05 by Student's *t*‐test.

BDNF produced by exercising skeletal muscle cells has been shown to activate AMPK (Pedersen *et al*., 2009) and it was recently demonstrated that AMPK regulates age‐related synapse remodelling of the mouse retina (Samuel *et al*., 2014). We therefore hypothesized that exercise‐mediated protection of the inner retina and maintenance of inner retinal synaptic integrity postinjury would be dependent on local or peripheral AMPK activation. To test this theory, we first conducted experiments in mice carrying a homozygous deletion of the β1 subunit of AMPK (AMPK β1−/−). These mice are viable, fertile and live for over 2 years with no obvious CNS abnormalities (Dzamko *et al*., 2010). As the retinal phenotype has not yet been described in this strain, we initially performed an analysis of baseline retinal structure and function in AMPK β1−/− mice. As expected, Fig. 5A shows that the AMPK β1 subunit is absent in retina of these mice and expression of the α1 catalytic subunit is significantly reduced, suggesting reduced AMPK activity. Histological analysis of retinal cross sections from 12‐month‐old AMPK β1−/− mice revealed no overt structural abnormalities (Fig. 5B). Furthermore, the β1 deletion had no detectable effect on retinal cell survival (Fig. 5C). Retinal function was measured serially in AMPK β1−/− and WT mice up to 12 months of age. As shown in Fig. 5D there was no genotype‐ or age‐related effect on ERG signals derived from photoreceptors (a‐wave), bipolar cells (b‐wave) or RGCs (pSTR). Collectively, these findings suggest that local AMPK deficiency does not impair retinal function and structure in mice up to middle age under standard housing conditions.

We next investigated the effect of exercise on the retinal response to injury in AMPK β1−/− mice. Unexercised WT and KO mice showed a similar susceptibility to pressure‐induced retinal injury, as shown by reductions in pSTR amplitudes to approximately 50% of baseline values (Fig. 5E). Interestingly, exercise conferred identical levels of functional protection after injury in both AMPKβ1−/− and WT mice, with pSTR amplitudes maintained at 80–90% for both genotypes. These data suggest that direct AMPK activation in the retina is not required for exercise‐mediated protection.

### Skeletal muscle AMPK activation is not critical for protection with exercise

**Figure 6:**
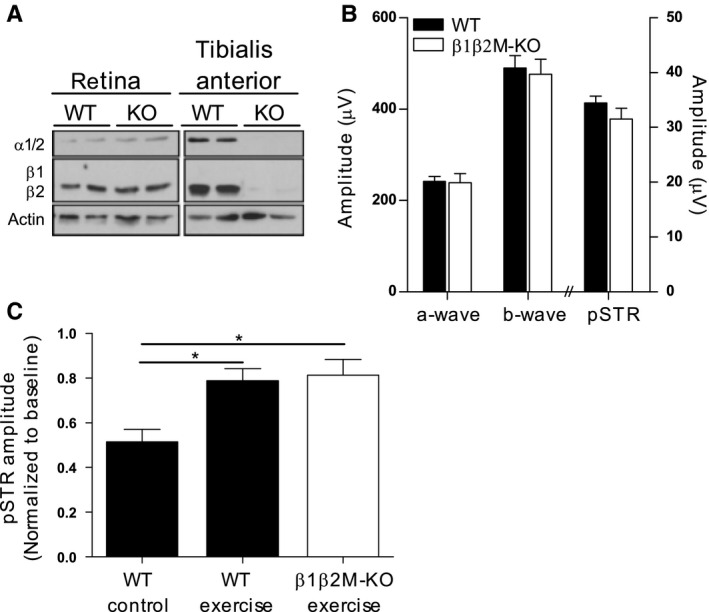
AMPK activation in skeletal muscle is not required for protection from injury with exercise. (A) Expression of AMPK α and β subunits was assessed in retina and tibialis anterior (TA) muscle of 12‐month‐old mice with muscle‐specific knock‐out of AMPK β1 and β2 subunits (β1β2M‐KO). (B) Baseline retinal function in KO and WT mice was assessed with the ERG (WT *n* = 12, KO *n* = 6). (C) Following pressure‐induced injury to the inner retina, animals were assigned to either exercise or control condition for 7 days. ERG amplitudes of the inner retinal‐derived pSTR waveform were measured in all mice before (baseline) and 7 days after injury and results are presented as relative changes from baseline. (WT *n* = 6, KO *n* = 6). Data are presented as mean ± SEM; *P < 0.05 by Student's *t*‐test.

Because AMPK activity in AMPKβ1−/− mice is normal in skeletal muscle, where the β2 subunit is predominant (Dzamko *et al*., 2010; Steinberg *et al*., 2010), we would anticipate maintenance of any muscle‐derived responses to exercise in this mouse strain. This led us to hypothesize that exercise‐induced retinal protection may be conferred indirectly by factors produced and secreted by contracting skeletal muscle cells (myokines). Recent reports show a critical role for AMPK in stimulating the expression, release and action of myokines (Lally *et al*., 2015; Lee *et al*., 2015). We therefore tested our hypothesis using a mouse strain that has muscle‐specific knock‐out of both AMPK β1 and β2 subunits (β1β2M‐KO). These mice have no detectable AMPK activity in skeletal muscle and are characterized by dramatic reductions in metabolic responses of muscle to exercise such as contraction‐stimulated glucose uptake (O'Neill *et al*., 2011). Figure 6A shows that protein expression of AMPK subunits β2, α1, α2 was absent in tibialis anterior muscles taken from β1β2M‐KO mice, confirming muscle‐specific AMPK deficiency. In contrast, retinal expression of β2, α1, α2 subunits was normal in β1β2M‐KO mice as expected. We compared retinal function between β1β2M‐KO and WT mice up to 12 months of age and found that ERG signals derived from outer, middle and inner retinal neurons were similar between genotypes (Fig. 6B). These results indicate that AMPK deficiency in muscle does not impair normal retinal function in mice at rest.

We next tested whether AMPK activation in skeletal muscle is critical for exercise‐mediated retinal protection. Although it has been reported that β1β2M‐KO mice have reduced tolerance for treadmill running (O'Neill *et al*., 2011), we did not encounter any difficulties in swimming these mice; exercise performance of β1β2M‐KO mice was indistinguishable from WT littermates. In response to pressure‐induced retinal injury, exercise conferred identical levels of protection in β1β2M‐KO and WT littermates (Fig. 6C), indicating that activation of AMPK in skeletal muscle is not required for retinal protection by exercise.

## Discussion

These data provide new insight into the mechanisms underlying exercise‐mediated protection of retinal cells. We found that daily forced exercise, initiated 24 h after an acute RGC‐specific injury in middle‐aged mice, led to a substantial improvement in RGC function and survival. Improved function was associated with reduced complement labelling and maintenance of the postsynaptic marker PSD‐95 in the inner plexiform layer of the retina. The protective effects of exercise were subsequently found to be critically dependent on BDNF signalling but independent of local and peripheral activation of AMPK. Whereas BDNF levels were significantly reduced in injured retinas of nonexercised mice, BDNF levels were maintained in injured retinas of exercised animals. Our findings support a model in which exercise promotes neuronal function and survival in the retina by maintaining levels of BDNF in injured RGCs and preventing complement‐mediated synapse elimination.

Synapse and dendrite remodelling has previously been reported in experimental animal models of glaucoma, with a significant reduction in the dendritic field radius, length and branching patterns prior to RGC death (Jakobs *et al*., 2005). Similar changes in dendrite bifurcation and branching patterns have also been reported in human glaucoma eyes (Stevens *et al*., 2007). Although we did not formally assess dendritic structure in our model, we found significant thinning of the IPL and loss of postsynaptic protein labelling following an acute injury that induced low levels of RGC loss. We went on to show that the structural and functional losses observed after injury were almost completely suppressed by exercise. Mice that began exercising the day after injury maintained normal inner retinal function 7 days postinjury compared with nonexercised animals, which lost 50% of inner retinal function. This finding implies that exercise preserves RGC function through maintenance of synaptic integrity. The minimal cell loss we see with injury in the absence of exercise cannot explain the accompanying 40–50% reduction in function, giving further strength to our hypothesis that synaptic changes are a key determinant of RGC functional loss in this injury model. Importantly, this implies that injured RGCs can be rescued from a period of functional loss by therapeutic or lifestyle interventions such as exercise.

The molecular mechanisms that drive synaptic remodelling in disease or injury are not known, but our data support a role for the complement cascade in the process. Activation of the classical complement cascade and deposition of complement proteins has been demonstrated in retinal degenerative disease in human postmortem tissue and in experimental animal models (Stasi *et al*., 2006; Kuehn *et al*., 2008; Tezel *et al*., 2010; Ding *et al*., 2012). We saw marked deposition of the complement protein C1q in mouse retinas following injury that colocalized with damaged synapses of the IPL, implicating the factor in retinal synapse loss. This mirrors a report by Stevens *et al*. (2007) where deposition of C1q in the IPL was seen in retinas from mice with inherited glaucoma. Indeed, there is growing evidence that complement proteins mediate elimination of CNS synapses by selectively binding and tagging inappropriate or defective synapses for pruning and elimination. This process has mainly been described during brain development but may also be aberrantly reactivated under neurodegenerative conditions (reviewed in Stephan *et al*., 2012). Exercising our mice postinjury almost completely blocked synaptic complement deposition, raising the possibility that this may be directly linked to preservation of synapses and the corresponding protection against dysfunction and loss of retinal neurons. Howell *et al*. (2011) showed that genetic C1q deficiency in a mouse model of glaucoma conveyed significant protection against RGC degeneration and death. Inhibiting the complement cascade may therefore serve as a neuroprotective therapy and exercise could be a noninvasive means of doing so.

It was recently reported that AMPK signalling regulates age‐related synaptic remodelling in the outer retina (Samuel *et al*., 2014); however, our data show that the ability of exercise to preserve inner retinal cell function after injury is not dependent on local or peripheral AMPK activation. This could imply distinct mechanisms of synapse maintenance in response to injury vs. aging and may also reflect the different structure and function of photoreceptor synapses of the outer retina vs. those of the inner retina.

Although retinal BDNF levels did not change in response to exercise alone, our data clearly identify a role for BDNF signalling and local BDNF protein in mediating protective effects of exercise after injury. RGCs have multiple, spatially distinct sources of BDNF that must be considered in this context. Endogenous BDNF is produced by RGCs themselves; neighbouring Müller cell glia produce and secrete BDNF; and exogenous BDNF is retrogradely transported along RGC axons from the optic tectum of the midbrain. Our current data show that almost all RGCs contained BDNF protein under normal conditions but that levels were dramatically reduced 7 days after injury. In stark contrast, BDNF protein was abundant in injured retinas from mice that had exercised postinjury, suggesting that exercise may promote cell survival and function by maintaining endogenous BDNF in RGCs. Despite maintenance of BDNF protein in exercised injured retinas, we did not detect a concurrent increase in BDNF mRNA. This may be attributed to the fact that BDNF protein levels can be independent of mRNA levels. It also raises the intriguing possibility that retinal BDNF protein in response to exercise originates from an external source. A probable candidate is the brain, which contributes 70–80% of circulating BDNF both at rest and during exercise (Rasmussen *et al*., 2009). BDNF may enter the retina from blood by crossing the blood–brain barrier or it may be retrogradely transported by RGC axons from their target areas in the brain. We are currently investigating these potential pathways.

Our findings are consistent with a theory posed by Vecino *et al*. (2002) that RGC survival after injury does not depend on the presence or absence of BDNF *per se* but on there being sufficient levels of BDNF in cells postinjury. In support, it is well known that exogenous delivery of BDNF to the eye can promote RGC survival after trauma. However, failure of exogenously applied BDNF to yield sustained protection means that translation of its neuroprotective potential has had little success. The transient nature of BDNF‐mediated protection is highlighted in our current study, where prevention against cell loss and synaptic thinning appeared to be lost once mice stopped exercising. Overcoming neurotrophin deprivation with regular exercise may therefore be an effective strategy for sustaining RGC integrity long term.

The contribution and relative importance of peripherally vs. locally produced BDNF to the neuroprotection afforded by exercise remains unclear. Similar to previous studies in the field, the two methods we used to block BDNF signalling (and block the accompanying neuroprotection) resulted in systemic BDNF depletion, thus preventing identification of the critical BDNF source. Increased BDNF protein and gene expression is well described in contracting skeletal muscle cells and was confirmed in our experiments, making BDNF an attractive candidate as a myokine for mediating beneficial effects of exercise. However, release of BDNF from contracting muscle into the circulation is yet to be shown. BDNF is also induced in various regions of the brain with exercise, most robustly in the hippocampus. Accordingly, we hypothesize that retinal protection with exercise may depend on BDNF supplied by the brain. While Lawson *et al*. (2014) reported a significant increase in retinal BDNF protein in mice exercised on a treadmill, we were unable to detect any change in retinal BDNF protein or gene expression with swimming exercise alone. This discrepancy may be due to the use of young mice; different types and durations of exercise; or the time elapsed between cessation of exercise and BDNF assessment. In skeletal muscle, exercise‐induced increases in BDNF expression are known to be dose and time dependent (Cuppini *et al*., 2007; Ogborn & Gardiner, 2010).

What are the links between synaptic remodelling and BDNF signalling? And what is the mechanism by which BDNF supports RGC survival and function after injury? It is possible that BDNF plays a direct role in maintaining retinal synaptic integrity after injury. In other regions of the CNS, BDNF is essential for supporting neuronal plasticity through diverse actions on axonal and dendritic remodelling, synaptogenesis and synaptic efficacy (Greenberg *et al*., 2009). While similar effects are yet to be characterized in the adult retina, BDNF is known to influence the morphological differentiation of RGC dendrites and axons during development (Cohen‐Cory & Lom, 2004). Synapse maintenance and remodelling require significant energy expenditure and BDNF may also provide metabolic support to these processes. Previous studies show that BDNF can function in a metabotrophic capacity to regulate energy metabolism and expenditure (Gomez‐Pinilla *et al*., 2008). Unravelling the mechanistic links between exercise‐induced retinal protection and BDNF signalling will be a key topic of future research.

## Experimental procedures

### Animals

All procedures conformed to the requirements of the Royal Victorian Eye & Ear Hospital Animal Research and Ethics Committee. Mice were housed in a temperature (22 ± 1 °C)‐ and light (12‐h light, 12‐h dark)‐controlled environment where food and water were available *ad libitum*. Male and female mice were used equally. Wild‐type and BDNF+/− mice on a C57BL/6 background were a kind gift from Marten van den Buuse and originated from a breeding colony at the Florey Institute of Neuroscience and Mental Health, Melbourne VIC, Australia. Wild‐type, AMPK β1−/− and β1β2M‐KO mice on a C57BL/6 background originated from a breeding colony at the St Vincent's Institute of Medical Research and Department of Medicine, Melbourne VIC, Australia. The generation and characterization of BDNF and AMPK knock‐out mice has been described previously (Ernfors *et al*., 1994; Dzamko *et al*., 2010; O'Neill *et al*., 2011).

### Exercise protocols

At 12 months of age, mice were randomly assigned to one of two experimental groups: exercise (swimming, 60 min day^−1^) or control (exercise handling procedure, 60 min day^−1^) as described (Chrysostomou *et al*., 2014). To minimize potential confounding influences of stress or environmental enrichment, mice in control groups were placed in an empty swimming tank alongside exercising mice for a comparable period each day and enrichment objects were excluded from all home cages.

### Elevation of intra‐ocular pressure

RGC injury was induced by short‐term elevation of intra‐ocular pressure, a well‐characterized nonischaemic injury that has been described in detail (Crowston *et al*., 2015).

### Electroretinography

The full‐field flash electroretinogram (ERG) was recorded using an Espion Diagnosys system as previously described (Chrysostomou & Crowston, 2013). In brief, animals were anaesthetized with intraperitoneal injection of ketamine (60 mg kg^−1^) and xylazine (10 mg kg^−1^). Pupil dilation and corneal anaesthesia were achieved by topical application of one drop each of tropicamide (0.5%), phenylephrine (2.5%) and proxymetacaine hydrochloride (0.5%). Electrical signals were recorded with a 4 mm‐platinum wire loop electrode contacting the cornea while a gold pellet placed in the mouth served as common reference. A subdermal needle electrode inserted at the base of the tail acted as ground. Retinal responses to a series of stimulus intensities (−5.92 to 2.22 log cd.s m^−2^) were recorded simultaneously from both eyes. ERG waveforms derived from different populations of retinal neurons were analysed as described (Chrysostomou & Crowston, 2013).

### Immunohistochemistry

Retinal crysosections were prepared from paraformaldehyde‐fixed eyes and subsequently immunolabelled according to the published protocols (Chrysostomou *et al*., 2014). The primary antibodies used were postsynaptic density protein‐95 (PSD‐95, 1:100; Invitrogen Camarillo, CA, USA), C1q (1:100; Abcam, Cambridge, MA, USA) and BDNF (1:200; Santa Cruz, Dallas, TX, USA).

### ANA‐12 administration

ANA‐12, *N*‐[2‐[[(Hexahydro‐2‐oxo‐1H‐azepin‐3‐yl)amino]carbonyl]phenyl]‐benzo[b]thiophene‐2‐carboxamide (Sigma‐Aldrich Pty. Ltd. Sydney, Australia), was dissolved in saline containing 1% DMSO. Mice received intraperitoneal injection of ANA‐12 (0.75 mg kg^−1^) or vehicle 60 min before commencing exercise or control conditions. The timing of ANA‐12 administration was selected to ensure peak bioavailability and TrkB inhibition during each exercise session (Cazorla *et al*., 2011).

### Quantification of retinal thickness and ganglion cells

The thicknesses of cellular and synaptic retinal layers were measured on digital images of Hoechst‐stained cryosections as described (Chrysostomou & Crowston, 2013). To quantify RGCs, sections cut through the optic nerve head and ora serrate were scanned from superior to inferior edge and the numbers of Hoechst‐labelled nuclei in the ganglion cell layer were counted by a masked observer.

### BDNF protein quantification

Immediately after the final exercise session, retina, soleus muscle and serum were collected from euthanized mice. Retina and muscle were placed into lysis buffer (1% Triton X‐100, 158 mm NaCl, 5 mm EDTA, 10 mm Tris [pH 7.0], 0.1% sodium orthovanadate, 0.1% aprotinin and 2% phenylmethylsulfonyl fluoride). Samples were sonicated and insoluble material was removed by centrifugation at 18 000 g for 20 min. Protein levels of BDNF were determined by ELISA (Biosensis RapidTM kit, Thebarton SA, Australia) according to the manufacturer's instructions.

### Quantitative PCR

Immediately after the final exercise session, retina and soleus muscle were dissected from euthanized mice and immersed in RNAlater (Sigma‐Aldrich Pty. Ltd. Sydney, Australia) for 24 h at 4 °C. RNA was isolated from tissues with QIAGEN RNeasy Mini kit (QIAGEN, Germantown MD, USA), and complementary DNA was subsequently synthesized using Applied Biosystems High Capacity RNA‐to‐cDNA kit. Quantitative real‐time PCR was performed using a TaqMan‐based assay (Applied Biosystems, Carlsbad CA, USA) with β‐actin as an endogenous reference. Relative quantitation of gene expression was calculated using the comparative threshold cycle method.

### AMPK immunoblotting

Tissues were homogenized with an electrical homogenizer (Tibialis anterior) or pellet pestle (retina) in ice‐cold lysis buffer containing 50 mm HEPES pH 7.4, 150 mm NaCl, 10 mm NaF, 1 mm sodium pyrophosphate, 0.5 mm EDTA, 250 mm sucrose, 1 mm dithiothreitol, 1% Triton X‐100, 1 mm Na_3_VO_4_ and 1 Roche protease inhibitor cocktail tablet per 50 mL of buffer. Protein concentration of the lysate was determined using the bicinchoninic acid method (Pierce, Thermo Fisher Scientific, Scoresby VIC, Australia). Lysate protein was resolved by SDS‐PAGE and detected by immunoblotting using primary antibodies for AMPK pan‐alpha and AMPK pan‐beta and pan‐actin (Cell Signaling, Danvers MA, USA). Protein was detected by enhanced chemoluminescence after incubation with a horseradish‐conjugated secondary antibody (Dako Australia Pty. Ltd. NSW, Australia).

### Statistical analysis

Paired data from control and exercise animals were assessed by a two‐tailed Student's *t*‐test. Comparisons within each treatment group were made by a one‐way analysis of variance with the Tukey post‐test used to compare group means. For all analyses, means were considered statistically different at *P* < 0.05. Statistical analyses were performed using GraphPad Prism 5.03 (GraphPad Software Inc., San Diego, CA, USA).

## Funding

The Ophthalmic Research Institute of Australia, (Grant/Award Number: ‘RANZCO Eye Foundation Grant’, ‘WA Quinlivan/Glaucoma Australia Grant’) National Health and Medical Research Council, (Grant/Award Number: ‘Project Grant APP1068813’). GRS is supported by a Canada Research Chair in Metabolism and Obesity and the J Bruce Duncan Chair in Metabolic Diseases.

## Conflict of interest

None declared.
